# Use of Digital Infrared Thermal Imaging in the Electromyography Clinic: A Case Series

**DOI:** 10.7759/cureus.4087

**Published:** 2019-02-18

**Authors:** Sameer S Ali, Arjumond Y Khan, Shon G Michael, Pavan Tankha, Hajime Tokuno

**Affiliations:** 1 Neurology, Veterans Affairs Hospital - Connecticut Healthcare System, West Haven, USA; 2 Neurology, Riverhills Neuroscience, Norwood, USA; 3 Pain Management, Veterans Affairs Hospital - Connecticut Healthcare System, West Haven, USA

**Keywords:** emg, denervation, electromyography, irt, foot drop, dorsiflexor, muscle atrophy, infrared thermography, peripheral nerve, complex regional pain syndrome (crps)

## Abstract

Introduction: Foot drop often results from denervation of the dorsiflexor muscles in the leg. Neurological evaluation begins with lower extremity motor testing followed by electromyography needle electrode examination (EMG-NEE). We explored digital infrared thermography (IRT) as a complementary tool in diagnosing peripheral nerve disorders.

Methods: Using a digital IRT camera, we recorded differences in skin surface temperatures from affected and unaffected limbs in three patients with unilateral foot drop. Denervation in the affected limb was confirmed with EMG-NEE.

Results: IRT imaging revealed lower relative skin surface temperatures in regions of the leg corresponding to denervated dorsiflexor muscles for all three consecutive patients who presented to the EMG Clinic with foot drop.

Conclusions: Denervation appears to cause a decrease in thermal energy output from affected muscle groups. Alongside the EMG and magnetic resonance imaging (MRI), IRT may have an important role in assessing the severity and prognosis of a nerve injury. This observation may have implications for chronic pain syndromes, such as complex regional pain syndrome (CRPS), in which thermal change is a diagnostic criterion.

## Introduction

This case series aims to demonstrate through the evaluation of three patients with unilateral foot drop that there may be a significant role for infrared thermography (IRT) as an adjunctive tool in diagnosing peripheral nerve syndromes. Specifically, it may help to more accurately detect the degree of axonal denervation and identify it earlier in its time course, therefore, representing a more sensitive diagnostic approach than current conventional methods. There may be potential for IRT to help us better understand and treat various neurologic conditions and pain syndromes.

Patients complaining of foot drop frequently present with varying degrees of paralysis in the ankle and toe dorsiflexor muscles. The neurological evaluation typically begins with lower extremity motor strength testing and electromyography (EMG) needle electrode examination (NEE) with or without nerve conduction studies (NCS). However, this approach has been shown to have significant limitations. NEE has only moderate to low sensitivity for detecting axonal denervation in muscles [1-2] and the reliability of test results can be undermined by poor patient cooperation, inter-observer variability, and inadequate sampling of needled sites [3-6].

Following the example of several academic institutions [7-8], we have assembled a mobile infrared thermography (IRT) unit as a routine clinical assessment tool in our Neurology Department at the Veterans Affairs (VA) Connecticut Healthcare Services. For the past year, we have used our IRT device, in conjunction with EMG and magnetic resonance imaging (MRI), to assist us in the clinical interpretation of a wide variety of peripheral nervous system disorders.

The IRT camera can measure heat emission from the surface of any solid object. IRT is a non-invasive, radiation-free measurement that provides objective quantitative data quickly and painlessly. The technology has been available to medical professionals for more than two decades, and it has been used by neurologists, pain anesthesiologists, oncologists, and physiatrists in numerous clinical settings [9-13]. For example, IRT has been used as an adjunct tool for diagnosing complex regional pain syndrome (CRPS) because CRPS patients nearly always present with decreased temperature in the affected limb [14]. Another group of investigators discovered a correlation between magnetic resonance imaging (MRI) findings and thermographic data in patients with lumbar radiculopathy symptoms. They reported decreased skin temperature over the multifidus muscles on the same side where the MRI demonstrated a lumbar nerve root compression [15]. Al Nakhli et al. used IRT to measure the thermal output from muscles during and after vigorous exercise among elite athletes [16]. They applied thermography to predict delayed onset muscle soreness in the quadriceps muscles.

## Materials and methods

The three patients in this case series were referred to the Neurology Service for evaluation of a unilateral foot drop. They were individually examined by a neuromuscular specialist in the EMG Clinic at the West Haven Campus of the VA Connecticut Healthcare Services between July and September 2015. A clinical interview and neurological examination preceded every IRT scan and EMG study.

**Figure 1:**
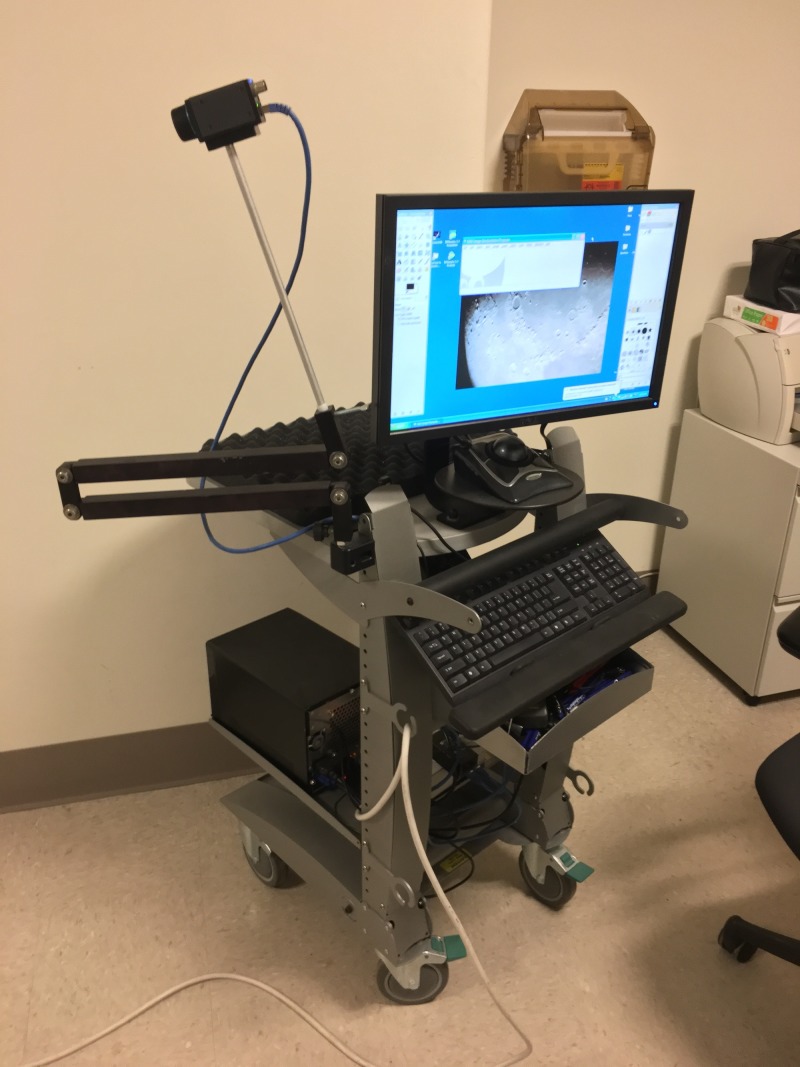
Mobile Infrared Thermography (IRT) Unit The flexible arm allows the examiner to position the camera at the appropriate distance and angle relative to the patient.

To acquire IRT images, we utilized the FLIR® A65sc camera (FLIR Systems Inc., Wilsonville, OR), which is a research-grade IRT image acquisition device with a resolution of 640 × 512 pixels. It is mounted atop a multijointed, anodized aluminum arm and interfaced to an ASUS laptop computer (AsusTeK Computer, Inc., Fremont, CA) via a category (CAT)-6 ethernet cable (Figure 1).

**Figure 2:**
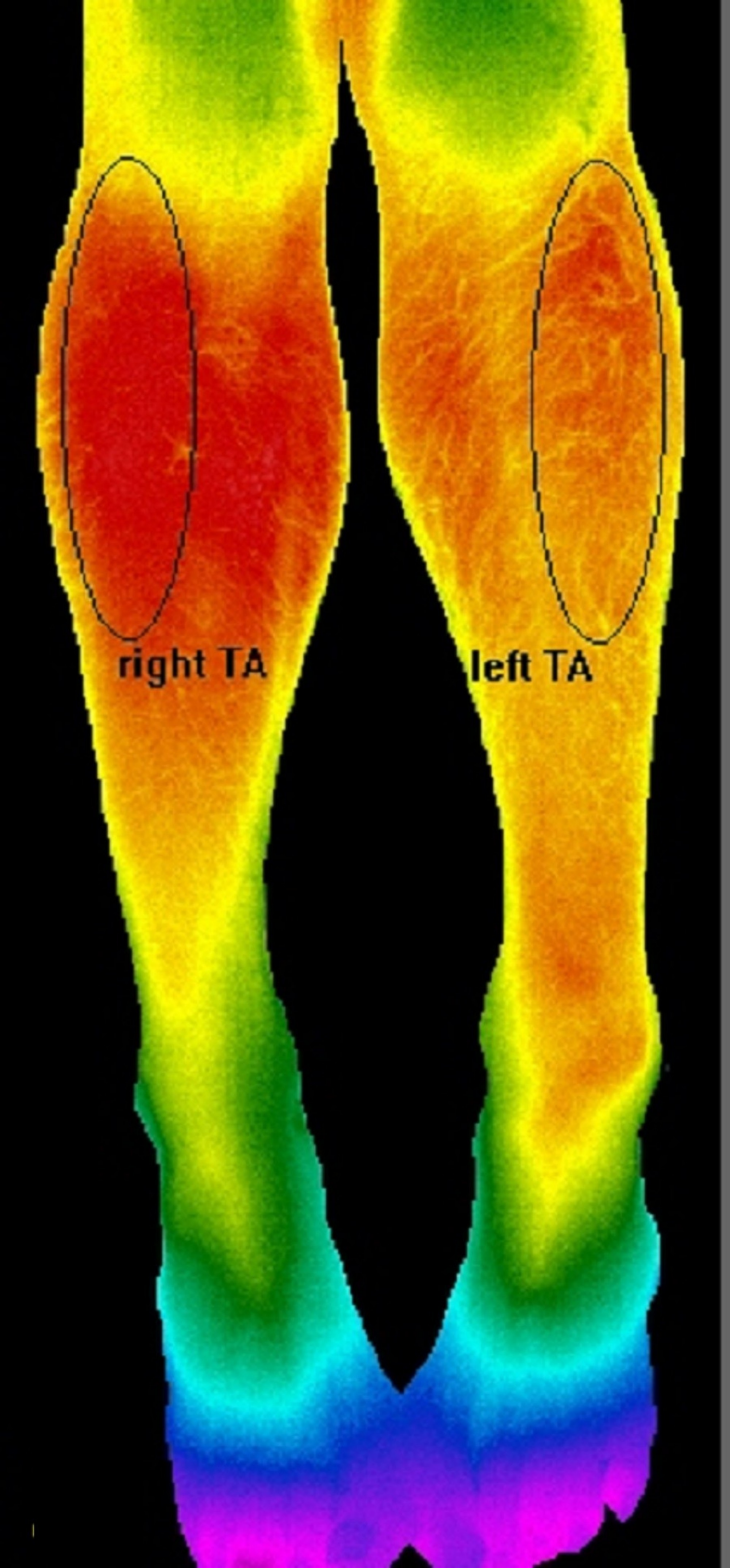
Anterior Compartment

**Figure 3:**
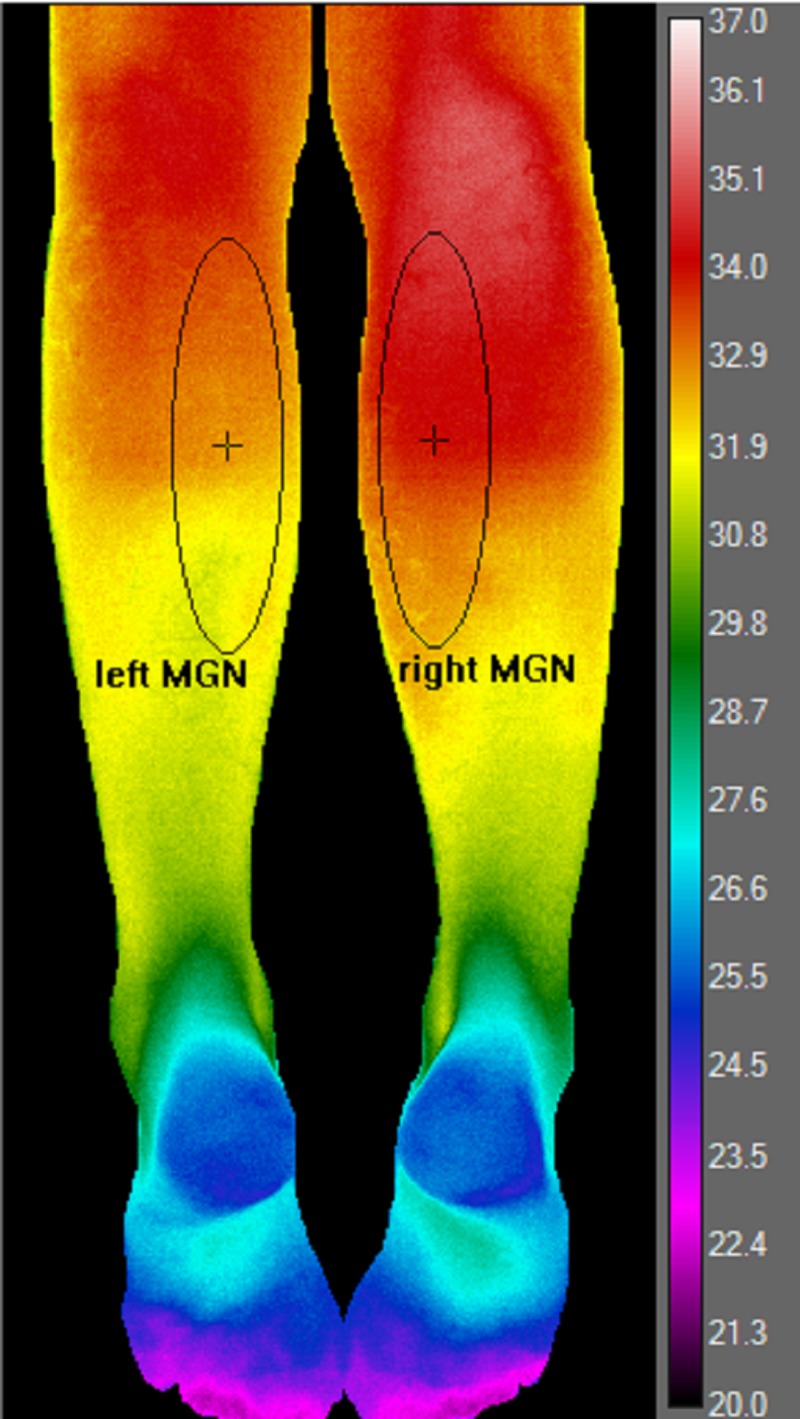
Posterior Compartment

The thermal sensitivity of the camera is < 0.05°C and its accuracy is ± 1.5°C at 30°C. Its spectral range is 7.5 – 13 micrometers, which are the wavelength appropriate for human body temperature measurements. In accordance with recommendations from FLIR technical support specialists, we powered up the camera and waited at least 30 minutes to allow the digital sensor (i.e., bolometer) to stabilize. After the patient acclimated to the ambient room temperature (20°C) for at least 15 minutes, a false color image of both legs was acquired in a single snapshot. To measure the signal from the tibialis anterior and other anterior compartment muscles, patients were placed in a supine position on the examination bed with both feet internally rotated 15-20 degrees as seen in Figure 2. For the medial and lateral gastrocnemius muscles, patients were placed in a prone position on the examination bed with their legs straight and relaxed (Figure 3).

Temperature measurements from the weak leg were compared to that of the unaffected leg, which served as an internal control. Data acquisition was performed using ResearchIR Max^©^, the proprietary software from FLIR Systems, Inc., Wilsonville, OR. We pre-selected several regions of interest (ROI) by highlighting the main belly of either anterior or posterior compartment muscles. We designed a software program that randomly selected 400 data points within each ROI. The ROI for the anterior compartment muscles circumscribed the belly of three muscles responsible for dorsiflexion: tibialis anterior (TA), extensor digitorum longus (EDL), and extensor hallucis longus (EHL) (Figure 2).

The vertical diameter of the ellipse-shaped ROI extended from the tibial tuberosity to the midpoint of the tibial shaft. The horizontal diameter of the ellipse encompassed the lateral upper quadrant of the leg as viewed from the front. Even though the anterior compartment muscles extend as far down as the dorsum of the foot, we did not record temperatures in the lower half of the leg (which is mostly tendon) [17]. In the posterior compartment, we created an elliptical ROI around the belly segment of the medial gastrocnemius (MGN) muscle (Figure 3).

For the MGN, the vertical diameter of the ellipse extended from the popliteal fossa to the midpoint of the tibial shaft. The horizontal diameter encompassed the medial upper quadrant of the leg viewed posteriorly. We did not include data from the lateral gastrocnemius muscle because its margins overlap with those of the peroneus longus, which has a different peripheral nerve and nerve root distribution. 

We used an unpaired, two sample, two-tail, Student’s t-test to probe for statistical significance in mean temperatures between the affected and unaffected muscle groups in each leg. We also calculated the effect size to estimate the magnitude of the temperature difference between the control and denervated legs. We used the absolute value of Glass’ Δ to approximate the effect size:

$&Delta; = (x ̅_1-x ̅_2)/s_2$

Where x̅_1_ is the mean temperature in the denervated limb, x̅_2_ is the mean temperature in the control limb and s_2_ is the standard deviation of the control. The cutoff ranges for Δ have been designated as follows: 0.2 (small), 0.4 (moderate), 0.6 (large), and 0.8 and above (very large) [18].

Electrophysiological studies to confirm the presence of denervation potentials were performed using a Dantec® EMG platform (2013 model) (Dantec Dynamics, Ltd., Bristol, United Kingdom). EMG-NEE was performed only after IRT scanning was completed in order to avoid temperature fluctuations from dry needling. At least four muscles in the weak limb were assayed for signs of denervation to distinguish between the L5 nerve root and peroneal nerve damage: the tibialis anterior (peripheral nerve: peroneal nerve, nerve root: L4/*L5*), the medial gastrocnemius (peripheral nerve: tibial nerve, nerve root: L5/*S1*), the tibialis posterior (TP) (peripheral nerve: tibial nerve, nerve root: *L5*/S1), and the lumbar paraspinal muscles (L4, L5, or S1, depending on the level tested). The italicized nerve root is the dominant segment, according to Tsao et al. [19].

## Results

Patient 1

This 68-year-old Caucasian male patient complained of a two-month history of left-sided foot drop. Motor examination showed a weakness of left foot dorsiflexion (4-/5 power) in the setting of normal plantar flexion, as well as normal leg extension and flexion (5/5). IRT scan showed significantly reduced temperature in the left anterior compartment muscles (Table 1A). Temperature reduction was also observed in the left MGN and the posterior compartment in general (Table 1B). The effect size, however, was more pronounced around the anterior compartment muscle bellies (Glass’ Δ = 2.3, very large effect) than around the MGN (Glass’ Δ = 0.2, small effect). NEE confirmed the presence of denervation potentials, decreased recruitment, and discrete interference pattern in the left TA and left TP muscles. The left MGN showed no spontaneous discharges, normal recruitment, and normal interference pattern. MRI of the lumbar spine showed moderate to severe bilateral neuroforaminal narrowing at the L4-L5 and L5-S1 levels. Based on the clinical findings, EMG results, and MRI report, we diagnosed the patient with probable left-sided L5 radiculopathy.

**Table 1:** Temperature Variations Between Control and Denervated Muscles of the Anterior and Posterior Compartment Muscles of the Lower Extremity

	1A. Anterior Compartment Muscles				1B. Posterior Compartment Muscles			
	Mean temperature (°C)		T-test	ES($\Delta$)	Mean temperature (°C)		T-test	ES($\Delta$)
	Control	Denervated	(p-value)		Control	Denervated	(p-value)	
Patient 1	34.7 ± 0.3	33.9 ± 0.3	< 0.0001	2.4	34.1 ± 0.3	34.0 ± 0.4	< 0.0001	0.2
Patient 2	33.9 ± 0.4	32.5 ± 0.3	< 0.0001	3.6	33.8 ± 0.2	33.3 ± 0.2	< 0.0001	0.8
Patient 3	32.8 ± 0.3	33.7 ± 0.5	< 0.0001	1.7	34.8 ± 0.7	33.9 ± 0.7	< 0.0001	1.3

Patient 2

The second patient was a 68-year-old Caucasian male with diabetes, recurrent pancreatic cancer, and Stage 3 squamous cell lung carcinoma who was referred to the EMG lab because of a five-week history of right-sided foot drop. Review of his chemotherapy regimen revealed that he had received several cycles of 5-fluorouracil and oxaliplatin. On motor examination, he showed 3/5 power in right dorsiflexion and 5/5 power in right plantar flexion. On IRT, we observed a significantly reduced temperature in the right anterior compartment muscles (Table 1A). A significant temperature reduction was also observed over the right MGN (Table 1B). The effect size was much greater in the anterior compartment muscles (Glass’ Δ = 3.6) than in the right MGN (Glass’ Δ = 0.8), but both effect sizes were large. NEE showed denervation potentials, decreased recruitment, and a discrete interference pattern in the right TA. No spontaneous discharges, decreased recruitment, or mixed interference patterns were noted in the tibialis posterior and left MGN. The MRI showed mild neuroforaminal narrowing at the L5/S1 level and no evidence of central canal stenosis. Given the relatively unremarkable MRI results, L5 radiculopathy due to nerve root compression was deemed unlikely. The differential diagnosis for the foot drop included metastatic infiltration of the peroneal or sciatic nerve, diabetic mononeuropathy multiplex, lumbar radiculitis, and chemotherapy-induced toxicity. 

Patient 3

This 58-year-old Caucasian man with a history of alcohol dependence was admitted to the medical inpatient unit for treatment of alcohol withdrawal. The patient reported that he had noticed a foot drop on his left side that appeared a week and a half earlier. He recalled that he had been drinking heavily when he tripped, fell, and injured his left ankle. On clinical exam, he exhibited flaccid paralysis of left ankle dorsiflexion (1/5 power) and trace weakness in left plantar flexion (4+/5 power). Leg extension and flexion were intact. IRT revealed a temperature decrease in the left anterior compartment muscles, as well as the left MGN muscles. Large effect sizes were noted in the temperature for both anterior and posterior compartments. The left TA revealed a markedly decreased recruitment and discrete interference pattern; the left TP showed moderately decreased recruitment and a mixed interference pattern. The left MGN showed normal recruitment. MRI of the lumbar spine was unremarkable. NEE showed no spontaneous discharges in all tested muscles. A repeat study was performed two weeks later at which time we documented the appearance of spontaneous discharges in the left TA. The differential diagnosis included subacute peroneal nerve injury and a distal sciatic nerve contusion from the fall. The trace weakness of the plantar flexors may not have been due to nerve injury but rather mechanical damage at the ankle.

## Discussion

Using infrared thermography, we measured reduced heat emissions from the denervated limbs of three patients with foot drop. We postulate that the decreased radiant energy is the result of diminished metabolic processes in muscles idled by axonal injury - regardless of location. All of our IRT images suggest that the region of maximum temperature coincided topologically with the belly of the largest muscles in the area.

Thermogenesis in humans, whether obligatory or adaptive, is mostly the product of oxidative phosphorylation and adenosine triphosphate (ATP) consumption within skeletal muscle tissue [20-23]. In addition, mitochondrial uncoupling proteins may augment heat production in myocytes under certain stressful conditions [24].

Studies in human and animal subjects have demonstrated that chronic denervation leads to a loss in muscle density and fiber size [25-27], as well as a reduction in capillary density and muscle perfusion [28]. At the cellular level, investigators have reported decreased mitochondrial content and apoptosis among myocytes [25, 29], as well as increased proteolysis and decreased glycolysis [30]. Therefore, as denervated muscles undergo degradative changes, they are likely to generate less energy and dissipate less heat compared to intact, healthy muscle tissues.

We also observed that the motor examination and needle electrode study of the MGN did not agree with IRT images in two out of three of the patients with foot drop. Whereas the motor exam and NEE showed normal responses, the IRT in all three patients revealed temperature reductions in the MGN muscles on the same side as the foot drop. We propose two possible explanations for this outcome: 

1) Despite normal strength and normal EMG findings in the plantar flexion muscles, subclinical denervation may be present in the gastrocnemii that is detectable only by IRT. Therefore, IRT may represent a more sensitive diagnostic approach compared to the motor exam or NEE.

2) Following the second law of thermodynamics, there may be thermal conduction (or heat flow) from the posterior to anterior compartment due to the temperature gradient created by the juxtaposition of intact, warmer muscles and denervated, cooler muscles.

Our case report is only a small sample and may not be representative of a larger population. A greater number of patients will have to be studied in order to confirm our findings. Moreover, this report describes cases involving only unilateral foot drop. It does not take into account patients with bilateral symptoms. In the next phase, a prospective study may be designed in which a large number of patients with new-onset foot drop can be followed over a longer period of time with EMG, IRT, and motor examination.

## Conclusions

In conclusion, we propose that infrared thermography may have an important role in evaluating muscle denervation when used in conjunction with an EMG needle study and MRI. Specifically, IRT may be utilized to assess the severity of denervation in a muscle, thereby better predicting the prognosis of nerve injury. IRT may provide insights not afforded by EMG in a variety of neurological disorders that involve axonal loss, such as mononeuritis multiplex, chronic inflammatory demyelinating polyneuropathy, traumatic neuropathies, and amyotrophic lateral sclerosis. Denervation appears to cause a decrease in thermal energy output from the affected muscle groups. This observation may have implications for chronic pain syndromes, such as complex regional pain syndrome, in which thermal change is a diagnostic criterion. 
